# Reduction of RKIP expression promotes nasopharyngeal carcinoma invasion and metastasis by activating Stat3 signaling

**DOI:** 10.18632/oncotarget.3847

**Published:** 2015-04-15

**Authors:** Qiu-Yan He, Hong-Mei Yi, Hong Yi, Ta Xiao, Jia-Quan Qu, Li Yuan, Jin-Feng Zhu, Jiao-Yang Li, Yuan-Yuan Wang, Li-Na Li, Juan Feng, Shan-Shan Lu, Zhi-Qiang Xiao

**Affiliations:** ^1^ Research Center of Carcinogenesis and Targeted Therapy, Xiangya Hospital, Central South University, Changsha, Hunan, China; ^2^ The Higher Educational Key Laboratory for Cancer Proteomics and Translational Medicine of Hunan Province, Xiangya Hospital, Central South University, Changsha, Hunan, China

**Keywords:** nasopharyngeal carcinoma, metastasis, RKIP, Stat3, metastatic suppressor

## Abstract

The role and underlying mechanism of Raf kinase inhibitory protein (RKIP) in nasopharyngeal carcinoma (NPC) metastasis remain unclear. Here, we showed that RKIP was downregulated in the NPC with high metastatic potentials, and its decrement correlated with NPC metastasis and poor patient survival, and was an independent predictor for reduced overall survival. With a combination of loss-of-function and gain-of-function approaches, we observed that high expression of RKIP reduced invasion, metastasis and epithelial to mesenchymal transition (EMT) marker alternations of NPC cells. We further showed that RKIP overexpression attenuated while RKIP knockdown enhanced Stat3 phosphorylation and activation in NPC cells; RKIP reduced Stat3 phosphorylation through interacting with Stat3; Stattic attenuated NPC cell migration, invasion and EMT marker alternations induced by RKIP knockdown, whereas Stat3 overexpression restored NPC cell migration, invasion and EMT marker alternations reduced by RKIP overexpression. In addition, there was an inverse correlation between RKIP and phospho-Stat3 expression in the NPC tissues and xenograft metastases. Our data demonstrate that RKIP is a metastatic suppressor and predictor for metastasis and prognosis in NPC, and RKIP downregulation promotes NPC invasion, metastasis and EMT by activating Stat3 signaling, suggesting that RKIP/Stat3 signaling could be used as a therapeutic target for NPC metastasis.

## INTRODUCTION

Nasopharyngeal carcinoma (NPC) is one of the most common malignant tumors in southern China and Southeast Asia [1]. Early metastasis is one of distinctive characteristics of NPC [2]. Although NPC is sensitive to radiotherapy, significant rates of relapse and distant metastasis still occur after therapy, which has been recognized as a major cause for NPC lethality [3]. However, the molecular mechanism of NPC metastasis is not yet well-defined. To develop better diagnosis and treatment approaches, it is important to understand the molecular basis of NPC metastasis.

Raf kinase inhibitory protein (RKIP) was initially identified as phosphatidylethanolamine-binding protein in bovine brain [4]. It was later identified RKIP as a physiological inhibitor of Raf kinase, antagonizing Raf-1/MEK/ERK pathway [5]. Further exploration revealed that RKIP inhibits NF-κB [6] and GRK2 (G protein-coupled receptor kinase 2) signaling [7], and activates GSK-3β signaling [8]. The recent study has found that RKIP blocks Stat3 signaling pathway [9]. Accumulative studies have showed that loss or downregulation of RKIP enhances epithelial to mesenchymal transition (EMT), motility, invasion and metastasis of tumor cells through regulating these pivotal intracellular signaling cascades [10, 11]. Previous studies have demonstrated that RKIP inhibits metastasis in prostate [12-14], breast [15], ovarian [16], colorectal [17, 18], and gastric cancer [19], and melanoma [20], and its expression is predictive of clinical outcome: better outcome with higher expression [12-19]. Additionally, downregulation of RKIP has been shown to impact therapy through conferring tumor radioresistance [21] and chemoresistance [22]. Thus, RKIP is considered to be a metastasis suppressor protein [10, 11].

Stat3, known to be activated by cytokines, growth factors, and oncogenic proteins, is constitutively activated in a number of malignancies including NPC [23], and promotes cell survival, EMT, invasion and metastasis [24-26]. Stat3, a latent cytoplasmic transcription factor, upon phosphorylated activation, translocates to the nucleus and binds to specific regulatory elements that control gene expression [27]. It has been reported that Stat3 constitutive activation and nuclear localization are associated with NPC cell growth, cancer stem-like characteristics and metastasis [23, 28-30], and activated Stat3 is a target for anti-NPC drug discovery [29-31].

In our previous comparative proteomic study, RKIP was identified as a downregulated protein in NPC tissues compared to normal nasopharyngeal mucosal tissues [32]. In another study, we also found that RKIP downregulation is associated with NPC radioresistance, and it is a potential biomarker for predicting NPC radiosensitivity [33]. LI et al. reported that RKIP expression is negatively associated with the distant metastasis of NPC, and has a predictive value for its distant metastasis [34]. However, the role and mechanism of RKIP downregulation in NPC metastasis are still unclear, and need to be elucidated.

As mentioned above, activated-Stat3 is correlated with NPC invasion and metastasis [23, 28-30], and RKIP blocks Stat3 activation in prostate cancer cells [9]. Furthermore, there is an inverse association between RKIP and Stat3 expression in gastric cancer tissues, and the high expression of cytoplasmic RKIP and nuclear Stat3 is correlated with poor patient prognosis [35]. Therefore, RKIP downregulation promotes tumor invasion and metastasis possibly through activating Stat3 signaling, but it needs to be validated.

In this study, we examined RKIP expression in thirty normal nasopharyngeal mucosal tissues, one hundred and twenty-seven NPC tissues with different metastatic potential and twenty paired neck lymph node metastases, analyzed the association of RKIP expression with NPC metastasis and patient prognosis; determined the role of RKIP in NPC invasion and metastasis both *in vitro* and *in vivo*; and explored whether Stat3 signaling mediates the effects of RKIP on NPC invasion and metastasis.

## RESULTS

### Expression levels of RKIP are associated with the metastasis and prognosis of NPC

Although RKIP expression has been shown to be associated with the metastasis and patient outcome of human cancers, its information in NPC is scarce. Accordingly, we were interested in assessing the expression and clinical significance of RKIP in NPC biopsies. IHC was performed in a cohort of NPC tissues including 79 NPCs with metastasis (neck lymphonode and/or distant metastasis) and 48 NPCs without metastasis, as well as 20 paired neck lymphonode metastases (LNMs) and 30 normal nasopharyngeal mucosa (NNM). The clinicopathologic features of the patients used in the present study are shown in Table 1. We observed that RKIP expression was dramatically reduced in the NPCs relative to NNM, in the NPCs with metastasis relative to NPCs without metastasis, and was not detectable in the almost all LNMs (Figure 1A, Table 2). The relationship between clinicopathological variables and RKIP expression is shown in Table 3. As shown in this table, reduced RKIP expression was correlated with advanced clinical stage and high frequent lymphonode and distant metastasis. Kaplan-Meier analysis revealed that low RKIP level in NPC tissues significantly correlated with the markedly reduced patient overall survival (Figure 1B). A univariate Cox regression analysis showed that RKIP expression level, lymphonode and distant metastasis and TNM stage markedly affected the overall survival of NPC patients (Table 4). A multivariate Cox regression analysis confirmed that low RKIP expression was an independent predictor for reduced patient overall survival (Table 4). These results indicate the importance of RKIP expression level in the NPC metastasis and patient prognosis.

**Table 1:** The clinicopathological parameters of 127 patients with nasopharygeal carcinoma

Variable	No. of patients	%
**Gender**		
Male	92	72.44
Female	35	27.56
**Age(y)**		
≥48	57	44.88
<48	24	55.12
**Primary tumor(T) stage**		
T1-2	43	34.65
T3-4	84	65.35
**Lymph node(N) metastasis**		
N0	49	38.58
N1-3	28	61.41
**Distant metastasis(M)**		
M0	99	77.95
M1	28	22.05
**Clinical TNM stage**		
I-II	24	18.9
III-IV	103	81.1

**Table 2:** Expression of RKIP and phospho-Stat3 in 127 nasopharygeal carcinomas

	NNM	NPC without metastasis	NPC with metastasis	LNM
**RKIP**				
High(4-6)	25	31	29	2
Low(0-3)	5	17	50	18
**Phospho-Stat3**				
High(4-6)	7	18	48	19
Low(0-3)	23	30	31	1

NNM, normal nasopharyngeal mucosa; LNM, lymphonode metastases.

*p*< 0.01 or 0.05 by χ^2^ test, NNM *vs*. NPC; NPC without metastasis *vs*. NPC with metastasis; NPC with metastasis *vs*. LNM

**Table 3:** Association between RKIP expression and clinicopathological characteristics in 127 nasopharyngeal carcinomas

		Expression level		
Variable	n	Low(0-3)	High(4-6)	*P*
**Gender**				
Male	92	47	45	0.368
Female	35	21	14	
**Age(y)**				
<48	57	32	25	0.596
≥48	70	36	35	
**Primary tumor(T) stage**				
T1-2	43	25	18	0.881
T3-4	84	50	34	
**lymph node(N) metastasis**				
N0	49	18	31	0.003^#^
N1-3	78	50	28	
**Distant metastasis(M)**				
M0	99	48	51	0.032*
M1	28	20	8	
**Clinical TNM stage**				
I-II	24	8	16	0.028*
III-IV	103	60	43	

# *P*<0.01 by χ^2^ test, N0 *vs*.N1-3.

* *P*<0.05 by χ^2^ test, M0 *vs*.M1; TNM stage I-II *vs*. III-IV.

**Table 4:** Univariate and multivariate analyses of selected prognostic factors for overall survival using Cox regression model (N=127)

Variable	Overall survival			
	Univariate analysis		Multivariate analysis	
	*P*	HR (95% CI)	*P*	HR (95% CI)
**Gender**				
Male *vs*. Female	0.295	1.377(0.757-2.506)	0.547	0.815(0.420-1.584)
**Age(ys)**				
<48 *vs*. ≥48	0.056	1.679(0.988-2.856)	0.312	1.349(0.755-2.412)
**Primary tumor(T) stage**				
T1-2 *vs*.T3-4	0.086	1.659(0.931-2.957)	0.148	1.630(0.337-1.179)
**lymph node(N) metastasis**				
N0 *vs*.N1-3	0.001^#^	2.569(1.485-4.799)	0.493	0.779(0.381-1.592)
**Distant etastasis(M)**				
M0 *vs*.M1	0.000^#^	7.864(4.539-13.625)	0.000^#^	6.711(3.761-11.974)
**Clinical TNM stage**				
I-II *vs*. III-IV	0.001^#^	10.950(2.657-45.130)	0.009^#^	6.985(1.639-29.763)
**RKIP expression level**				
Low *vs*. high	0.000^#^	0.348(0.200-0.605)	0.000^#^	0.335（0.189-0.596）

# *P*<0.01

**Figure 1:**
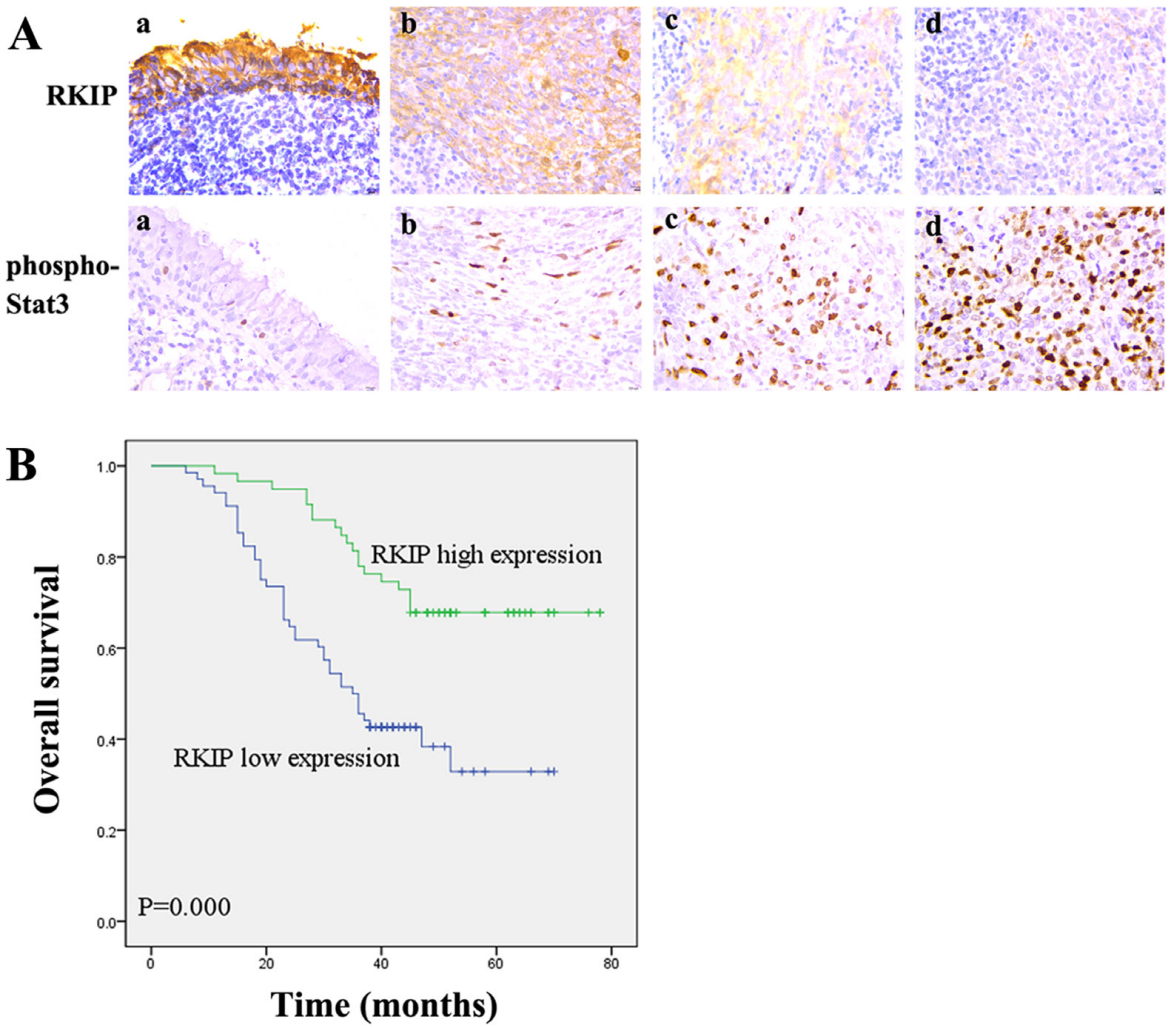
Association of RKIP expression levels with NPC metastasis and overall survival of the patients **A**. a representative result of RKIP and phospho-Stat3 immunohistochemical staining in normal nasopharyngeal mucosa (a), NPC without metastasis (b), NPC with metastasis (c), and lymphonode metastasis (d). Original magnification, ×200. **B**. Kaplan-Meier survival analysis for NPC patients according to the expression levels of RKIP. NPC patients with low RKIP expression have a significantly worse overall survival than those with high RKIP expression. The log-rank test was used to calculate p value.

### RKIP downregulation promotes NPC cell migration and invasion *in vitro*

**Figure 2:**
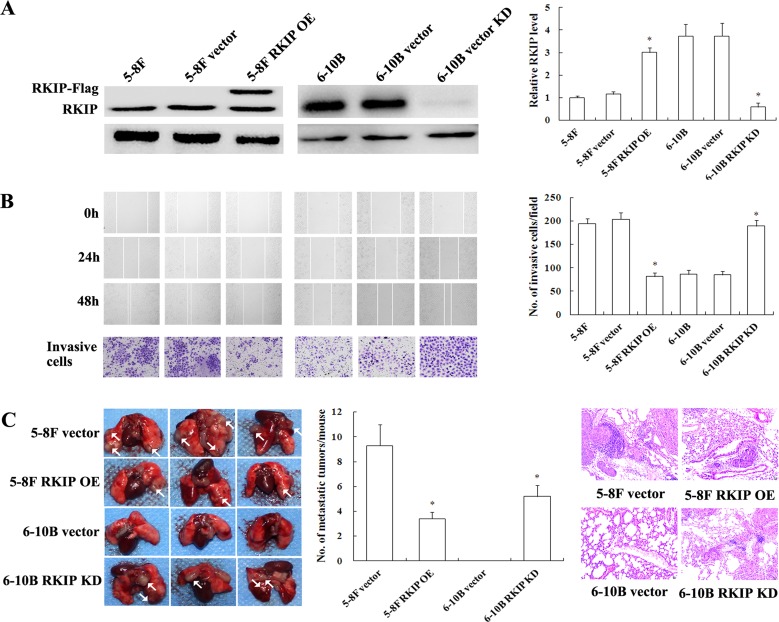
The effects of RKIP expression changes on *in vitro* NPC cells migration and invasion and *in vivo* metastasis **A**. (left) a representative result of Western blotting shows the levels of RKIP expression in 5-8F and 6-10B NPC cells and their transfectants; (right) histogram of relative RKIP expression levels in 5-8F and 6-10B NPC cells and their transfectants as determined by densitometric analysis. **B**. (left top) a representative result of scratch wound-healing assay shows migration of 6-10B and 5-8F cells and their transfectants. Images were taken at 0, 24 and 48h after wounding under the inverted microscope; (left bottom) a representative result of Matrigel invasion assay shows invasion of 6-10B and 5-8F cells and their transfectants. Invasive cancer cells were photographed at 48h after incubation; (right) histogram of average numbers of invasive cancer cells per microscopic field. **C**. *in vivo* metastasis assays of NPC cells with RKIP expression changes. (left) RKIP overexpression 5-8F cells, RKIP knockdown 6-10B cells, and their corresponding empty vector-transfected cells were injected into the tail vein of nude mice, and the representative photography of lung is shown; (middle) histogram of average numbers of lung metastases per mouse; (right) the representative H&E staining of lung tissues shows metastatic tumors. Metastases generated by RKIP overexpression 5-8F cells are significantly less than those generated by empty vector-transfected 5-8F cells. The empty vector-transfected 6-10B cells do not generate metastases, but RKIP knockdown 6-10B cells can generate metastases. Columns, mean values; bars, S.D. *, *p* < 0.01. Vector, transfcted with an empty vector; OE, overexpression; KD, knockdown.

To investigate the effect of RKIP in NPC cell migration and invasion *in vitro*, we established 5-8 NPC cell lines with stable overexpression of RKIP, 6-10B NPC cell lines with stable knockdown of RKIP, and their corresponding control cell lines (Figure 2A). As shown in Figure 2B, RKIP overexpression decreased while RKIP knockdown increased NPC cell migration and invasion as determined by Scratch wound-healing and Matrigel invasion assays. These results demonstrated that RKIP downregulation promoted NPC cell migration and invasion *in vitro*.

### RKIP downregulation promotes NPC cell metastasis *in vivo*

To explore the role of RKIP in NPC metastasis *in vivo*, we tested the effect of RKIP in a xenograft metastasis model in which RKIP knockdown 6-10B cells, RKIP overexpression 5-8F cells, and their corresponding control cells were used to generate pulmonary metastases in nude mice. As shown in Figure 2C, pulmonary metastases generated by RKIP overexpression 5-8F cells were significantly less than those generated by empty vector-transfected 5-8F cells. Moreover, although control 6-10B cells without metastatic potential did not generate pulmonary metastases, RKIP knockdown 6-10B cells could generate pulmonary metastases (Figure 2C). The results suggested that RKIP played a crucial role in inhibiting NPC metastasis, and its downregulation promoted NPC cell metastasis *in vivo*.

### RKIP downregulation induces EMT-like molecular alternations in NPC cells

**Figure 3:**
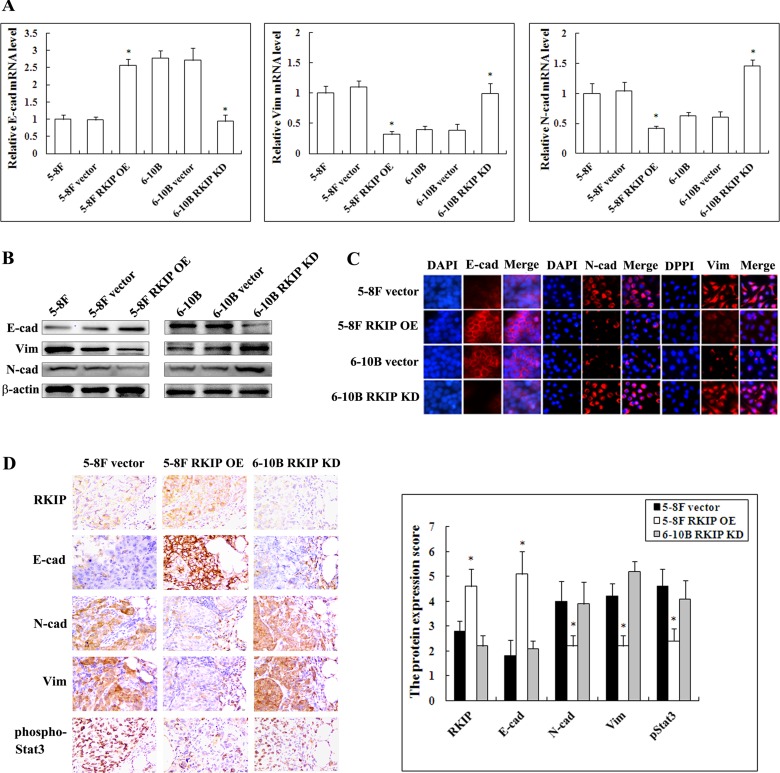
The regulation of RKIP on the expression of EMT-like cellular markers in NPC cells and their xenograft metastases **A**. mRNA expression levels of E-cadherin, N-cadherin and Vimentin in 5-8F and 6-10B NPC cells and their transfectants detected by qRT-PCR. Columns, mean values from triplicate experiments; bars, S.D. *, *p* < 0.01. **B**. a representative result of Western blotting shows the levels of E-cadherin, N-cadherin and Vimentin in 5-8F and 6-10B NPC cells and their transfectants. **C**. a representative result of immunofluorescent staining shows the levels of E-cadherin, N-cadherin and Vimentin in RKIP overexpression 5-8F cells, RKIP knockdown 6-10B cells and their corresponding empty vector-transfected cells. **D**. (left) a representative result of immunohistochemical staining of RKIP, E-cadherin, N-cadherin, Vimentin and phospho-Stat3 in the lung metastases of RKIP overexpression 5-8F cells, RKIP knockdown 6-10B cells and empty vector-transfected 5-8F cells; (right) histogram of expression levels of RKIP, E-cadherin, N-cadherin, Vimentin and phospho-Stat3 in the lung metastases. Original magnification, ×200. Columns, mean values from 10 mice; bars, S.D. *, *p* < 0.01. E-cad, E-cadherin; N-cad, N-cadherin; Vim, Vimentin. Vector, transfcted with an empty vector; OE, overexpression; KD, knockdown.

EMT is a key event in cancer invasion and metastasis [36]. Previous studies have indicated that RKIP overexpression inhibits EMT in prostate cancer cells [37, 38]. Therefore, we analyzed the effect of RKIP on the expression of representative EMT markers in NPC cells. QRT-PCR and Western blotting analyses showed that mesenchymal markers Vimentin and N-cadherin significantly upregulated while epithelial marker E-cadherin significantly downregulated in RKIP knockdown 6-10B cells compared with control 6-10B cells (Figure 3A and 3B). Conversely, Vimentin and N-cadherin significantly downregualted while E-cadherin significantly upregulated in RKIP overexpression 5-8F cells compared with control 5-8F cells (Figure 3A and 3B). Moreover, immunofluorescent staining also revealed that the similar expression pattern of Vimentin, N-cadherin and E-cadherin in RKIP knockdown 6-10B cells and RKIP overexpression 5-8F cells (Figure 3C). The results suggested that RKIP downregulation induced EMT in NPC cells.

Next, we detected the expressions of representative EMT markers in the xenograft metastases using IHC. The results showed that Vimentin and N-cadherin significantly downregulated while E-cadherin was significantly upregulated in the metastases of RKIP overexpressing-5-8F cells as compared to control 5-8F cells metastases (Figure 3D). Moreover, the expression levels of Vimentin, N-cadherin and E-cadherin in the metastases of RKIP knockdown 6-10B cells, parental cells of which lack metastatic potential, were comparable to those in the metastases of control 5-8F cells with high metastatic potential. These results support our *in vitro* findings. Taken together, the results implied that RKIP downregulation enhanced NPC cell invasion and metastasis possibly by inducing EMT.

### RKIP downregulation activates Stat3 signaling in NPC cells

**Figure 4:**
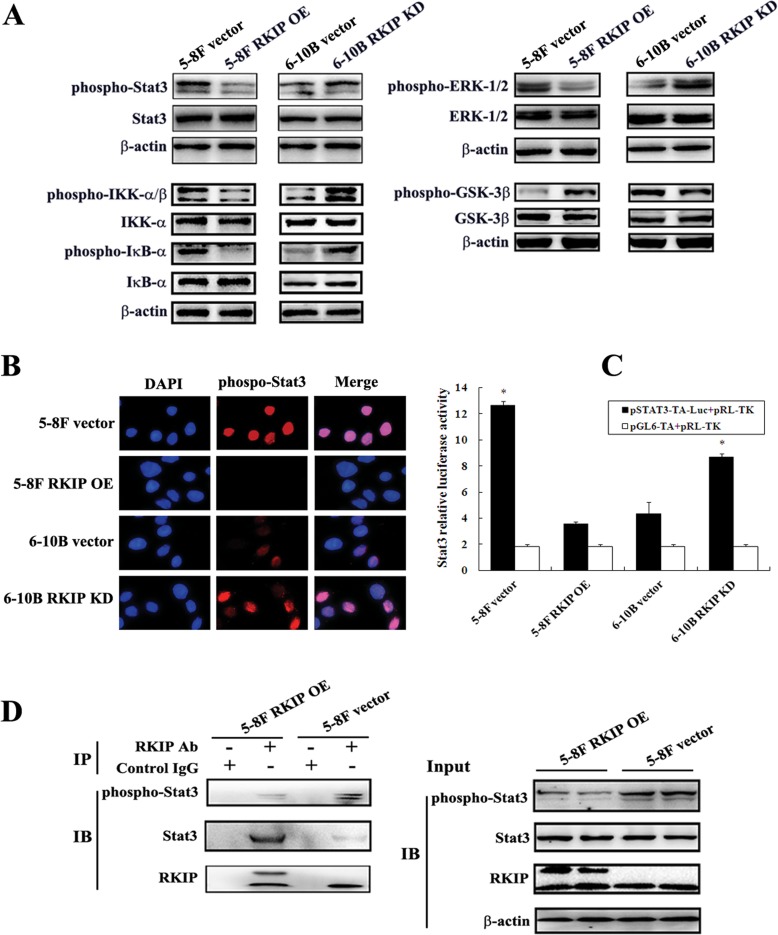
The regulation of RKIP on the activity of NPC cellular signaling pathways **A**. a representative result of Western blotting shows the phosphorylated and total levels of Stat3, ERK-1/2, IKK-α/β, IκB-α, and GSK-3β in RKIP overexpression 5-8F cells, RKIP knockdown 6-10B cells and their corresponding empty vector-transfected cells. **B**. a representative result of immunofluorescent staining shows the nuclear translocation of phospho-Stat3 in RKIP overexpression 5-8F cells, RKIP knockdown 6-10B cells and their corresponding empty vector-transfected cells. **C**. Stat3 luciferase reporter activity in RKIP overexpression 5-8F cells, RKIP knockdown 6-10B cells and their corresponding empty vector-transfected cells. Columns, mean values from triplicate experiments; bars, S.D. *, *p* < 0.01. **D**. a representative result of Co-IP shows RKIP interacting with and inhibiting Stat3 phosphorylation in RKIP overexpression 5-8F cells. Cells were lysed in RIPA buffer, a portion of the sample was removed as the IP input and the remaining supernatant was immunoprecipitated with RKIP antibody and protein A agarose. Immunocomplexes were separated by SDS-PAGE, transferred onto PVDF membrane, and detected with Stat3, phospho-stat3 or RKIP antibody. The input samples were examined for the expression of the indicated proteins. Vector, transfcted with an empty vector; OE, overexpression; KD, knockdown.

To determine the signaling mechanisms of RKIP-regulating NPC cell invasion and metastasis, we detected the effects of RKIP on the phosphorylated level of ERK-1/2, Stat3, NF-κB and GSK-3β by Western blotting. The results showed that the phosphorylated level of Stat3 (Tyr705), ERK-1/2 (Thr202/Tyr204), IKK-α (Ser176)/β(Ser177) and IκB-α (Ser32) obviously increased, and that of GSK-3β (Ser9) obviously decreased in RKIP knockdown 6-10B cells compared with control 6-10B cells. Conversely, the phosphorylated level of Stat3 (Tyr705), ERK-1/2 (Thr202/Tyr204), IKK-α(Ser176)/β(Ser177) and IκB-α (Ser32) obviously decreased, and that of GSK-3β (Ser9) oviously increased in RKIP overexpression 5-8F cells compared with control 5-8F cells (Figure 4A). The results indicated that RKIP inhibited ERK, NF-κB, and Stat3, and activated GSK-3β in NPC cells, which is consistent with previous reports [5, 6, 8, 9]. Although RKIP can block Stat3 activity [9], it is unknown whether Stat3 mediates RKIP-regulating tumor cell invasion and metastasis. Therefore, we selected RKIP/Stat3 signaling for further study.

Once phosphorylated and activated, Stat3 enters the nucleus and regulates gene transcription [27]. Accordingly, we detected nuclear phospho-Stat3 expression in the NPC cells with RKIP overexpression or knockdown by immunefluorescent staining. We found that the number of cells expressing nuclear phospho-Stat3 is markedly increased in RKIP knockdown 6-10B cells, whereas obviously decreased in RKIP overexpression 5-8F cells compared with their corresponding control cells (Figure 4B), indicating that RKIP inhibited Stat3 nuclear translocation in NPC cells. We next detected the ability of RKIP to inhibit transcriptional activity of Stat3 using a luciferase reporter assay. The results showed that Stat3 luciferase reporter activity was remarkably increased in RKIP knockdown 6-10B cells, whereas remarkably decreased in RKIP overexpression 5-8F cells compared with their corresponding control cells (Figure 4C), demonstrating that RKIP was able to inhibit Stat3 transcriptional activity. Taken together, the results demonstrated that RKIP blocked Stat3 signaling in NPC cells.

It has been reported that RKIP overexpression resulted in constitutive physical interaction with Stat3 and blocked c-Src-phosphorylating and activating Stat3 [9]. Therefore, we detected whether RKIP interacts with, and inhibits Stat3 activation using immunoprecipitaion in NPC cells. We found that exogenous expression of RKIP in 5-8F cells not only increased RKIP-interacting Stat3, but also decreased phospho-Stat3 level compared with control cells, indicating that RKIP inhibited Stat3 activation also by interacting with Stat3 in NPC cells (Figure 4D).

### RKIP downregulation enhances migration, invasion, and EMT-like molecular alterations by activating Stat3 signaling in NPC cells

**Figure 5:**
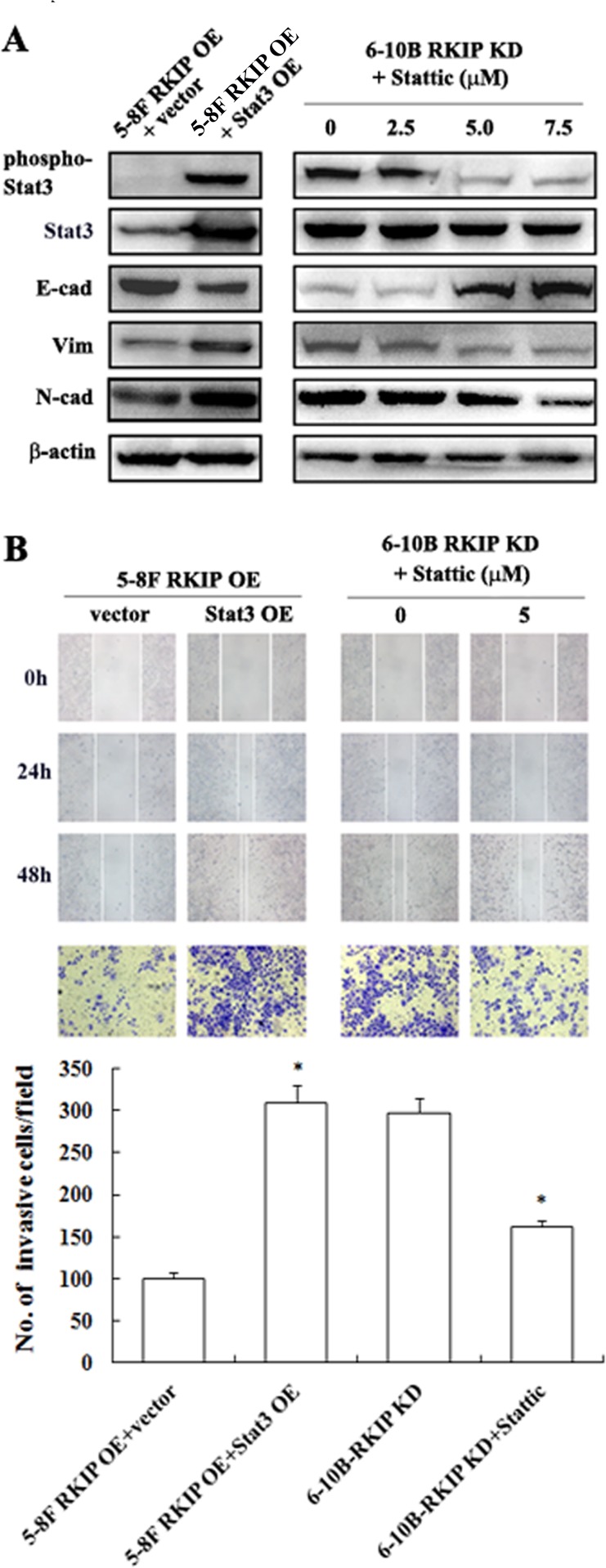
The regulation of RKIP on migration, invasion, and EMT marker expressions mediated by Stat3 signaling in NPC Cells **A**. a representative result of Western blotting shows the levels of E-cadherin, N-cadherin and Vimentin in RKIP overexpression 5-8F cells transfected with pReceiver-M13-Stat3 or empty vector pReceiver-M13, and RKIP knockdown 6-10B cells treated with a range 0-7.5 μM Stat3 inhibitor Stattic. **B**. a representative result of scratch wound-healing assay (top) and Matrigel invasion assay (middle) of RKIP overexpression 5-8F cells transfected with pReceiver-M13-Stat3 or pReceiver-M13, and RKIP knockdown 6-10B cells treated with Stat3 inhibitor Stattic; (bottom) histogram of average numbers of invasive cancer cells per microscopic field in these cells. Columns, mean values; bars, S.D. *, *p* < 0.01. Vector, transfcted with an empty vector; OE, overexpression; KD, knockdown.

Stat3 is constitutively activated in NPC cells [23], and activated Stat3 promotes NPC cell invasion, metastasis and EMT [28-30], which encouraged us to determine whether RKIP downregulation promotes migration and invasion, and induces EMT in NPC cells via activating Stat3 signaling. We observed that transfection of Stat3 restored EMT-like molecular alterations, and cell migration and invasion reduced by RKIP overexpression in RKIP overexpression 5-8F cells (Figure 5A and 5B, Figure 2B and 3B). Treatment of RKIP knockdown 6-10B cells with Stat3 inhibitor Stattic abolished EMT-like molecular alterations, and cell migration and invasion induced by RKIP knockdown (Figure 5A and 5B, Figure 2B and 3B). Collectively, these data demonstrated that Stat3 mediated RKIP-regulating NPC cell migration, invasion and EMT, and RKIP downregulation promoted NPC cell invasion, metastasis and EMT by activating Stat3 signaling.

Next, we determined whether the expression levels of RKIP and phospho-Stat3 were correlated in the cohort of NPC tissues and xenograft metastases using IHC. The results showed that phosph-Stat3 level was significantly higher in NPCs with metastasis than that in tumors without metastasis, and in lymphonode metastases than that in paired primary NPCs (Figure 1A; Table 1). Correlation analyses revealed that there was an inverse association between RKIP and phospho-Stat3 expression in the NPC tissues (*P* < 0.01, r = −0.585). Phospho-Stat3 expression was reduced in the xenograft metastases of RKIP overexpressing-5-8F cells compared with control 5-8F cells metastases (Figure 3D). Moreover, phospho-Stat3 expression level in the metastases of RKIP knockdown 6-10B cells was comparable to that of control 5-8F cells with high metastatic potential (Figure 3D). These results supported our *in vitro* findings, and suggested that RKIP downregulation might contribute to NPC cell metastasis *in vivo* by activating Stat3 signaling.

## DISCUSSION

In this study, we found that RKIP expression reduced in NPCs with metastasis as compared to NPCs without metastasis, and almost lost in lymphonode metastases. RKIP decrement in NPC correlated with high frequent metastasis, advanced clinical stage and poor patient survival, and was an independent predictor for reduced overall patient survival. To confirm the effects of RKIP downregulation on NPC cell invasion and metastasis, we established NPC cell lines with stable RKIP expression changes. We found that RKIP overexpression reduced while RKIP knockdown enhanced NPC cell invasion and metastasis both *in vitro* and *in vivo*. Moreover, RKIP knockdown 6-10B cells, parental cells of which lack metastatic potential, also produced pulmonary metastases in nude mice. Taken together, our results demonstrated that RKIP is a metastasis suppressor protein of NPC.

EMT is a key event in cancer invasion and metastasis [36]. Previous studies have indicated that RKIP inhibits EMT of prostate cancer cells [37, 38]. Therefore, we analyzed the effect of RKIP on the expression level of representative EMT markers in NPC cells. The results showed that RKIP knockdown increased Vimentin and N-cadherin expression while decreased E-cadherin expression in NPC cells. RKIP overexpression decreased Vimentin and N-cadherin expression while increased E-cadherin expression in NPC cells. Moreover, downregulation of mesenchymal markers while upregulation of epithelial marker were also observed in the xenograft metastases of RKIP overexpressing-5-8F NPC cells. The expression levels of Vimentin, N-cadherin and E-cadherin in the xenograft metastases of RKIP knockdown 6-10B cells were comparable to those in the metastases of 5-8F cells with high metastatic potential. These results indicated that RKIP downregulation induced EMT-like cellular molecular alterations in NPC cells and xenograft metastases, and RKIP downregulation promoted NPC cell invasive and metastatic capability possibly by inducing EMT.

To determine the signaling mechanisms of RKIP-regulating NPC cell invasion and metastasis, we firstly explored the effect of RKIP on the activity of ERK-1/2, NF-κB, GSK-3β and Stat3 signaling. The results showed that RKIP knockdown increased the phosphorylated levels of ERK-1/2, IκB-α, IKK-α and Stat3, whereas decreased phosphorylated GSK-3β level in NPC cells. RKIP overexpression had the opposite effect on the phosphorylated levels of these proteins in NPC cells. The results indicated that RKIP inhibited ERK, NF-κB, and Stat3 signaling, and activated GSK-3β signaling in NPC cells, which is consistent with the previous reports in the other types of cancers [5, 6, 8, 9].

It is unknown whether Stat3 mediates RKIP-regulating tumor cell invasion and metastasis. Therefore, we further investigated the effects of RKIP on Stat3 signaling in NPC cells. Immunofluorescent staining showed that RKIP knockdown increased while RKIP overexpression decreased the number of cells expressing nuclear phospho-Stat3 in NPC cells, indicating that RKIP could inhibit Stat3 nuclear translocation. Moreover, RKIP knockdown increased while RKIP overexpression decreased Stat3 luciferase reporter activity in NPC cells, demonstrating that RKIP could inhibit Stat3 transcriptional activity. Interestingly, we found although RKIP overexpression increased RKIP-binding Stat3, the phosphorylated level of RKIP-binding Stat3 was decreased in NPC cells, suggesting that RKIP inhibited Stat3 activity by interacting with Stat3 and then blocking its phosphorylation. Indeed, a recent study also showed that RKIP overexpression resulted in constitutive physical interaction with Stat3 and blocked c-Src-phosphorylated and activated Stat3 [9].

Given that activated Stat3 promotes invasion and metastasis in a number of malignancies including NPC cells [24-26, 28-30], we determined whether Stat3 mediates RKIP-inhibiting NPC cell invasion and metastasis. We observed that Stattic treatment reduced while exogenous expression of Stat3 enhanced *in vitro* tumor cell migration and invasion, as well as EMT-like molecular alterations in NPC cells. These results demonstrated that RKIP downregulation promoted NPC cell invasion, metastasis and EMT by activating Stat3 signaling. We also observed that phosph-Stat3 level was significantly higher in NPCs with metastasis than that in tumors without metastasis, and in lymph node metastases than that in paired primary NPCs. There was an inverse association between RKIP and p-Stat3 expression in NPC tissues. Moreover, phospho-Stat3 expression was reduced in the xenograft metastases of RKIP overexpressing-5-8F cells as compared to control metastases, and phospho-Stat3 expression level in the metastases of RKIP knockdown 6-10B cells was comparable to that of 5-8F cells with high metastatic potential. These results supported our *in vitro* findings, and suggested that RKIP downregulation might also contribute to *in vivo* NPC cell metastasis by activating Stat3 signaling.

In summary, our data demonstrate that: i) RKIP was downregulated in NPC cells and tissues with high metastatic potentials, and low RKIP expression was an independent predictor for poor overall survival of NPC patients; ii) RKIP downregulation promoted NPC cell invasion, metastasis and EMT-like molecular alterations both *in vitro* and *in vivo*; iii) RKIP inhibited Stat3 activation in NPC cells by interacting with and then blocking Stat3 phosphorylation in NPC cells; iv) RKIP downregulation promoted NPC invasion, metastasis and EMT-like molecular alterations by activating Stat3 signaling.

## MATERIALS AND METHODS

### Patients and tissue specimens

The one hundred and twenty-seven formalin-fixed and paraffin-embedded archival NPC tissue specimens (79 NPCs with metastasis and 48 NPCs without metastasis) between Jan 2007 and Dec 2009 were obtained from the First Hospital of Chengzhou City (Chengzhou, China) at the time of diagnosis before any therapy. In addition, 20 paired lymph node metastatic NPCs, and 30 normal nasopharyngeal mucosa were also collected. On the basis of the 1978 WHO classification [39], all tumors were histopathologically diagnosed as poorly differentiated squamous cell carcinomas (WHO type III). The clinical stage of all the patients was classified according to the 1992 NPC staging system of China [40]. All the patients underwent radio-chemotherapy treatment and were given follow-up. The follow-up period at the time of analysis was 6 to 78 months (average, 41 ±17.3). The total survival was defined as the time from diagnosis to the date of cancer-related death or when censured at the latest date if patients were still alive.

### Reagents, plasmids and cell lines

Anti-RKIP, Anti-phosph-Stat3 (Tyr705), anti-Stat3, anti-ERK-1/2 (Thr202/Tyr204), anti-ERK-1/2, anti-phospho-GSK-3β (Ser9), anti-GSK-3β, anti-phospho-IKK-α/β (Ser176)/β(Ser177), anti-IKK-α, anti-phospho-IκB-α (Ser32), anti-IκB-α and anti-E-cadherin antibodies were purchased from Cell Signaling Technology. Anti-Vimentin and anti-N-cadherin antibodies were purchased from Abcom. Horseradish peroxidase-conjugated goat anti-rabbit IgG and anti-mouse IgG antibodies were purchased from Life technologies. Fluorescence-conjugated second antibodies (DyLight 594-labeled anti-rabbit Ig and anti-mouse Ig) were purchased from Vector Laboratories. Stat3 kinase inhibitor Stattic was purchased from Merck. Recombinant plasmid pcDNA3.1(+)-Flag-RKIP and lentiviral pGV248-puro-RKIP shRNA were established by Genechem (Shanghai, China), and confirmed by sequencing. The targets for human RKIP shRNA were 5′-GGTGGCGTCCTTCCGTAAA-3′, the effect of which has been validated [41]. The shRNA targeting the 5′-TGGCTGCATGCTATGTTGA-3′ sequence served as a negative control. Recombinant plasmid pReceiver-M13-Stat3 and control vector pReceiver-M13 were purchased from GeneCopoeia. Stat3 luciferase reporter vector pSTAT3-TA-luc and control vector pGL6-TA were purchased from Beyotime (Nanjin, China). pRL-TK vector was purchased from Promega. High metastatic NPC 5-8F and non-metastatic NPC 6-10B cell lines were maintained in RPMI-1640 medium supplemented with 10% FBS, 100 U/ml penicillin, and 0.1 mg/ml streptomycin.

### Establishment of NPC cell lines with overexpression or knockdown of RKIP

To generate NPC cell lines with RKIP overexpression, RKIP expression plasmid pcDNA3.1(+)-Flag-RKIP and control plasmid pcDNA3.1(+) were transfected into non-metastatic 6-10B NPC cells using Lipofectamine 2000 (Invitrogen) according to the manufacturer's instruction, respectively. To generate NPC cell lines with RKIP knockdown, lentiviral pGV248-puro-RKIP shRNA and pGV248-puro-control shRNA were used to transduce high metastatic 5-8F NPC cells according to the manufacturer's instruction, respectively. Cells were selected using neomycin or puromycin for 2 weeks, and NPC cell lines with stable overexpression or knockdown of RKIP and control cell lines were obtained.

### Scratch wound-healing assay

Cell migration was determined by scratch wound-healing assay. Briefly, cells were grown in RPMI 1640 medium containing 10% FBS overnight to confluence in a 6-well plate. Monolayers of cells were wounded by dragging a pipette tip. Cells were washed to remove cellular debris and allowed to migrate for 24-48h. Images were taken at 0, 24 and 48h after wounding under the inverted microscope.

### Matrigel invasion assay

An invasion assay was performed in 24-well 8-mm pore size transwell chambers precoated with Matrigel (BD Biosciences) according to the manufacturer's instruction. The upper chamber was filled with 1×10^5^ cells in RPMI 1640 medium containing 0.5% FBS. The lower chamber was filled with RPMI 1640 medium containing 10% FBS as a chemoattractant. After incubation at 37°C for 48h, cells were fixed with 4% paraformaldehyde and stained with 0.5% crystal violet. Cells migrating through the Matrigel and the pores of the filter were counted from four random microscopic fields.

### Experimental lung metastasis in nude mice

Nude male mice that were 4 weeks old were obtained from the Laboratory Animal Center of Central South University (Changsha, China) and were maintained under specific pathogen-free conditions. For experimental lung metastasis, mice (*n* = 10 each group) were injected intravenously with 1×10^6^ cells/mouse via the tail vein. 6 weeks after injection, mice were sacrificed by cervical dislocation, and lungs were removed, weighed, and embedded in paraffin for hematoxylin and eosin (H.E.) and immunohistochemical staining. Lung metastases were examined macroscopically and detected in the paraffin-embedded tissue sections stained with H.E. staining.

### Quantitative reverse transcription-PCR

Total RNA was extracted from cells using Trizol reagent (Invitrogen). 2 μg of total RNA was reversely transcribed for cDNA using the reverse transcription (RT) kit (Promega) and Oligo dT primer according to the manufacturer's instruction. The RT products were amplified by real-time PCR using QuantiFast SYBR green PCR kit (Qiagen) according to the manufacturer's instruction, and GAPDH was used as the internal control to normalize the expression levels of different genes. qRT-PCR was performed on the ABI Gene Amp PCR System 9700 (ABI). The primers used for amplification of indicated genes are listed in Supplementary Table S1.

### Western blotting

Cells were lysed in RIPA buffer. An equal amount of protein in each sample was mixed with Laemmli buffer and subjected to sodium dodecyl sulfate-polyacrylamide gel electrophoresis (SDS-PAGE) separation, followed by blotting onto a polyvinylidene difluoride (PVDF) membrane (Millipore). Blots were blocked with 5% nonfat dry milk or 3% BSA for 2h at room temperature and then incubated with primary antibody overnight at 4C°, followed by incubation with horseradish peroxidase-conjugated secondary antibody for 1h at room temperature. The signal was visualized with an enhanced chemiluminescence detection reagent (Pierce). β-Actin was detected simultaneously using monoclonal mouse anti-β-actin antibody (Sigma) as a loading control.

### Immunohistochemistry

Immunohistochemical staining of RKIP and phospho-Stat3 (Try705) in the cohort of clinical NPC tissues and xenograft metastases was performed on formalin-fixed and paraffin-embedded tissue sections. Briefly, tissue sections were treated with an antigen retrieval solution [10 mmol/L sodium citrate buffer (pH 6.0)]; incubated with mouse monoclonal anti-RKIP antibody (1:800 dilution) (CST; #13006), or rabbit polyclonal anti-phospho-Stat3(Tyr705) antibody (1: 400) (CST; #9145) overnight at 4°C; and then were incubated with 1:1000 dilution of biotinylated secondary antibody followed by avidin-biotin peroxidase complex (DAKO). Finally, tissue sections were incubated with 3′, 3′-diaminobenzidine (Sigma) until a brown color developed and were counterstained with Harris' modified hematoxylin. In negative controls, primary antibodies were omitted.

The immunoreactions of RKIP and phospho-Stat3 were evaluated independently by two pathologists. Staining intensity was categorized: absent staining as 0, weak as 1, moderate as 2, and strong as 3. The percentage of stained cells was categorized as no staining = 0, <30% of stained cells = 1, 30~60% = 2, and >60% = 3. The staining score (ranging from 0-6) for each tissue was calculated by adding the area score and the intensity score. A combined staining score of ≤3 was considered to be low expression, and > 3 was considered to be high expression.

### Dual luciferase reporter assay

5×10^5^ cells were plated into 60 mm culture dish chamber and incubated with RPMI-1640 medium containing 10% FBS for 12h. Cells were transiently cotransfected with 0.5 μg of a reporter plasmid containing human STAT3 response element (pSTAT3-TA-luc) and 0.5 μg of pRL-TK plasmid using lipofectamine 2000 in serum-free medium. Cotransfection of pGL6-TA without STAT3 response element and pRL-TK plasmid into cells served as a control. Cells were harvested 48h after transfection, and both firefly luciferase and renilla luciferase activities were measured with the Dual-luciferase reporter assay system (Promega) according to the manufacturer's instruction, and transcriptional activity of Stat3 was estimated using a luminometer.

### Immunofluorescent staining

2×10^4^ cells were plated into chamber slides (Millipore) and cultured with RPMI-1640 medium containing 10% FBS for 12h. Cells were fixed with 4% paraformaldehyde at room temperature for 15min, and then cell membranes were permeabilized with 0.1% Triton 100 at room temperature for 20min. Cells were washed with 1×PBS and blocked with 10% goat serum in PBS for 1h. Then cells were incubated with primary antibodies overnight at 4°C. After washing with 1×PBS for three times, cells were incubated with secondary antibodies conjugated with Alexa Fluor 594 for 1h. The slides were washed three times with 1×PBS, counterstained with DAPI, mounted and stored at 4°C under dark conditions. Pictures were taken under a Leica DMI4000 microscope.

### Immunoprecipitation assay

The cells were lysed in RIPA buffer. 1.0 mg of proteins was incubated with 3 μg of monoclonal mouse-anti human RKIP antibody (Santa Cruz; sc-365973) for 4h at 4ºC. Immunocomplexes were collected on protein A-agarose beads (Life Technologies), separated by 10% SDS-PAGE and electroblotted to PVDF membrane. Proteins were detected after incubation with specific antibodies described and identified using an enhanced chemiluminescence detection reagent(Pierce). Five percent of the sample was removed to verify and analyze proteins present for the immunoprecipitation input.

### Statistical analysis

All experiments were carried out at least 3 times. Data were presented as the mean ± standard deviation (SD). Statistical analysis was conducted using SPSS18.0 software. For comparisons between two groups, a Student t test or chi-square test was used, and for analysis with multiple comparisons, Oneway ANOVA test was used. Survival curves were obtained by using Kaplan–Meier method, and comparisons were made by using log-rank test. Univariate and multivariate survival analyses were conducted on all parameters by using Cox proportional hazards regression model. The Spearman rank correlation coefficient was used to determine correlation between RKIP and p-Stat3 expression levels in the NPC tissues. *P* values less than 0.05 were considered to be statistically significant.

### Ethics statement

This study was approved by the Institute Research Ethics Committee of Central South University, China. Written informed consent was obtained from all participants in the study. All animal experiments were undertaken in accordance with the Guide for the Care and Use of Laboratory Animals of Central South University, with the approval of the Scientific Investigation Board of Central South University.

## SUPPLEMENTARY MATERIAL TABLE


