# Supplementary material for: Reduction of RKIP expression promotes nasopharyngeal carcinoma invasion and metastasis by activating Stat3 signaling
Source: Oncotarget. 2015 Apr 15;6(18):16422–36. doi: 10.18632/oncotarget.3847 (PMC4599279; doi:10.18632/oncotarget.3847)
Supplement: Supplementary file 1 [file oncotarget-06-16422-s001.pdf]

Reduction of RKIP expression promotes nasopharyngeal carcinoma invasion and metastasis by activating Stat3 signaling

Supplementary Material

Table S1. The primers used for the amplification of the five genes by qRT-PCR

No.	Gene name	GenBank Accession No.	Primer sequence	Product size(bp)
1	RKIP	NM_002567	F:CTACACCTTGGTCCTGACAGA<br>R: GAGCCCACATAATCGGAGAGG	118
2	E-cadherin	NM_004360	F: CTTAGAGGTCAGCGTGTGTG<br>R: AGCAAGAGCAGCAGAATCAG	147
3	Vimentin	NM_003380	F: AATGGCTCGTCACCTTCG<br>R: CTAGTTTCAACCGTCTTAATCAG	225
4	N-cadherin	NM_004061	F: CCACGCCGAGCCCCAGTATC<br>R: CCCCCAGTCGTTCAGGTAATCA	232
5	GAPDH	NM_002046	F: TGACTTCAACAGCGACACCCA<br>R: CACCCTGTTGCTGTAGCCAAA	121
